# Lamivudine plus darunavir boosted with ritonavir as simplification dual regimen in HIV-infected patients

**DOI:** 10.7448/IAS.17.4.19801

**Published:** 2014-11-02

**Authors:** Jose Luis Casado, Sara Bañón, Ana Moreno, Alberto Diaz de Santiago, Cristina Gomez, María J Perez-Elías, Santiago Moreno

**Affiliations:** Infectious Diseases, Ramon y Cajal Hospital, Madrid, Spain

## Abstract

**Introduction:**

The combination of lamivudine plus a protease inhibitor boosted with ritonavir (PI/r) has become an alternative as simplification strategy in HIV-infected patients with toxicity/intolerance to other nucleoside analogues (NA). Lamivudine plus darunavir/r (DRV/r) could be an adequate once daily option.

**Materials and Methods:**

Prospective cohort study of 48 HIV-infected patients on suppressive triple therapy-based HAART, HBV negative, who switched to lamivudine 300 mg plus DRV/r 800/100 mg once daily.

**Results:**

Mean age was 50 yrs (35–74), and 65% were male. Thirty patients (63%) had HCV co-infection (fibrosis 4 in 7 cases, 23%). Median time of HIV infection was 19.1 years, and CD4+ count nadir was 220 cells/µL (2–604). They had received a mean of three regimens before (2–20), and 20 (42%) had a previous AIDS diagnosis. In eight cases, a previous resistance test showed two to seven secondary mutations in the protease gene, without resistance to DRV/r (one patient with the I84V mutation). At baseline, patients had viral suppression (<50 copies/mL) for a median time of 1263 days (341–1884), and they were receiving predominantly a PI based regimen (ATV in four, FPV in four, LPV in three, DRV in six) or an efavirenz-based regimen (seven). The main reason to switching to this dual therapy was toxicity (35 patients, 75%), mainly renal toxicity attributed to tenofovir (24 cases). During 104.3 patients-year of follow-up (median 912 days), only two patients (4%) failed at 27 and 505 days, due to non-adherence and lost to follow up, respectively. Total cholesterol and triglycerides increased significantly during the first six months after initiation (TC, from 185 to 269 mg/dL; p=0.01, TG from 118 to 185 mg/dL; p=0.03, TC/HDL ratio, from 4.09 to 4.66) and decreased after. Median estimated glomerular filtration rate (eGFR) improved during follow up (from 86 to 96.1 mL/min; p=0.13). In patients with renal toxicity as cause of switch there was a mild, no significant improvement during the first year (from 63.3 to 68.7 mL/min), although an improvement in protein-uria levels (protein/creatinine ratio, from 171 to 126 mg/g; p=0.04) was observed soon after initiation.

**Conclusions:**

Dual therapy with lamivudine plus darunavir boosted with ritonavir once daily is safe and effective as simplification strategy in HIV-infected patients.

